# Pre- and post-bronchodilator lung function as predictors of mortality in the Lung Health Study

**DOI:** 10.1186/1465-9921-12-136

**Published:** 2011-10-12

**Authors:** David M Mannino, Enrique Diaz-Guzman, Sonia Buist

**Affiliations:** 1 University of Kentucky, College of Public Health, Lexington, KY, USA; 2 University of Kentucky College of Medicine, Lexington, KY, USA; 3 Oregon Health Sciences University, Portland, OR, USA

**Keywords:** COPD, mortality, epidemiology, bronchodilator responsiveness

## Abstract

**Background:**

Chronic obstructive pulmonary disease (COPD) is supposed to be classified on the basis of post-bronchodilator lung function. Most longitudinal studies of COPD, though, do not have post-bronchodilator lung function available. We used pre-and post bronchodilator lung function data from the Lung Health Study to determine whether these measures differ in their ability to predict mortality.

**Methods:**

We limited our analysis to subjects who were of black or white race, on whom we had complete data, and who participated at either the 1 year or the 5 year follow-up visit. We classified subjects based on their baseline lung function, according to COPD Classification criteria using both pre- and post-bronchodilator lung function. We conducted a survival analysis and logistic regression predicting death and controlling for age, sex, race, treatment group, smoking status, and measures of lung function (either pre- or post-bronchodilator. We calculated hazard ratios (HR) with 95% confidence intervals (CI) and also calculated area under the curve for the logistic regression models.

**Results:**

By year 15 of the study, 721 of the original 5,887 study subjects had died. In the year 1 sample survival models, a higher FEV_1 _% predicted lower mortality in both the pre-bronchodilator (HR 0.87, 95% CI 0.81, 0.94 per 10% increase) and post-bronchodilator (HR 0.84, 95% CI 0.77, 0.90) models. The area under the curve for the respective models was 69.2% and 69.4%. Similarly, using categories, when compared to people with "normal" lung function, subjects with Stage 3 or 4 disease had similar mortality in both the pre- (HR 1.51, 95% CI 0.75, 3.03) and post-bronchodilator (HR 1.45, 95% CI 0.41, 5.15) models. In the year 5 sample, when a larger proportion of subjects had Stage 3 or 4 disease (6.4% in the pre-bronchodilator group), mortality was significantly increased in both the pre- (HR 2.68, 95% CI 1.51, 4.75) and post-bronchodilator (HR 2.46, 95% CI 1.63, 3.73) models.

**Conclusions:**

Both pre- and post-bronchodilator lung function predicted mortality in this analysis with a similar degree of accuracy. Post-bronchodilator lung function may not be needed in population studies that predict long-term outcomes.

## Background

COPD is a chronic disease of the lungs and is characterized by irreversible airflow limitation, and is currently the third leading cause of death in the United States [1-3]. GOLD defines COPD as a preventable and treatable disease with airflow limitation that is usually progressive and associated with an abnormal inflammatory response of the lung to noxious particles or gases [4]. Both the American Thoracic Society (ATS) and the European Respiratory Society (ERS) have, in large part, adopted this definition [5].

Response to a bronchodilator is thought to be important in COPD diagnosis and guidelines suggest that classification of COPD be made using spirometry performed after bronchodilator administration [4]. While asthma generally has more reversibility to a bronchodilator than COPD, the presence of reversibility does not distinguish asthma from COPD [6].

According to the 2008 GOLD guidelines "Spirometry should be performed after the administration of an adequate dose of an inhaled bronchodilator (e.g., 400 μg salbutamol) [7] in order to minimize variability. In a random population study to determine spirometry reference values, post-bronchodilator values differed from pre-bronchodilator values [8]. Furthermore, post-bronchodilator lung function testing in a community setting has been demonstrated to be an effective method to identify individuals with COPD [9,10]. However, most longitudinal studies looking at the effect of impaired lung function on outcomes such as mortality and hospitalizations have used pre-bronchodilator lung function [11-14].

The purpose of this study is to determine whether pre- or post-bronchodilator lung function differentially predict mortality in cohorts over time. Data from the Lung Health Study [15] was used in this analysis.

## Methods

The Lung Health Study (LHS) was a randomized multicenter clinical trial that was carried out from October 1986 through April 1994 [15,16]. A detailed description of the LHS design has been previously published [16]. Briefly, "healthy" current smokers between the ages of 35 and 60 were enrolled if their forced expiratory volume in one second (FEV_1_) to forced vital capacity (FVC) was less than 70% and their FEV_1 _was between 55% and 90% of the predicted normal value. Subjects were randomized into three groups: a control group receiving "usual care", a smoking intervention group receiving placebo, and a smoking intervention group receiving the bronchodilator ipratroprium. Lung function was measured before and after two inhalations (200-μg total dose) of isoproteronol from a metered-dose inhaler.

We used data from the year 1(one year following baseline) and year 5 (5 years following baseline) visits and included subjects who had complete data and both pre- and post-bronchodilator lung function measurements at these visits. The rationale for using these visits was that the inclusion criteria limited the range of lung disease severity at baseline to mild and moderate COPD, whereas a broader range could be seen in subsequent visits. In addition, prior work has demonstrated that bronchodilator responsiveness was larger in year 1 and subsequent years than it was at baseline [17].

About 75% of the original cohort of 5887 participants were followed continuously for 10 years beyond the 5-year time frame of Lung Health Study I (these subjects were mostly participants in Lung Health Study III) by biannual phone contacts (to ascertain vital status, smoking status, morbidity and mortality). Our primary endpoint was all-cause mortality at up to 14.5 years of follow-up from baseline [18]. The time metric used was time from the year 1 examination to the time of death or the end of the study or from the year 5 examination to the end of the study.

### Study Measures

Predicted values from the Third National Health and Nutrition Examination Survey (NHANES III) were used in the analysis [19]. We used age, sex, height and race to determine the predicted values. The study participants were classified, using the pre- and post-bronchodilator lung function, into five lung function categories based on a modification of COPD classification criteria: Normal (no airflow obstruction or restriction), restricted (FEV_1_/FVC ≥ 70% and FVC < 80% of predicted), Stage 1 (FEV_1_/FVC < 70% and FEV_1 _≥ 80% predicted), Stage 2 (FEV_1_/FVC < 70% and 50% ≤ FEV_1 _< 80% predicted), and Stage 3 or 4 (FEV_1_/FVC < 70% and FEV_1 _< 50% predicted) [4].

### Definitions

Demographic data included in this analysis were sex, age, body mass index (BMI), smoking status, race, and educational status. Age was classified at baseline, the year 1, and year 5 examinations and was categorized for use in tables (35-39, 40-49, 50-50, and 60 and older), and was used as a continuous variable in the survival analyses. BMI was categorized at baseline and was categorized into 3 categories (< 25, 25-29, and > = 30 kg/m^2^), and was used as a continuous variable in the survival analyses. All subjects were smokers at baseline, so smoking status was classified based on their second through fifth follow-up visits as current smokers for those who never stopped smoking, former smokers for those who successfully quit, and intermittent smokers for those whose status varied [20]. Education status was stratified into three levels (< 12 years, 12 years, and > 12 years). Race was classified as White or Black, with people of other races excluded. The original design of the study was incorporated by stratifying the subjects by randomization group: Intervention with ipratroprium, Intervention with placebo, and Control.

### Statistical Analysis

Data analysis was completed using statistical software (Statistical Analysis Software, version 9.2; SAS Institute; Cary, NC and SUDAAN version 10.1; RTI, Research Triangle Park, NC). Our primary outcome of interest in the survival models was mortality, and the main predictor of interest in our analysis was COPD severity defined by stage of lung function, both pre- and post-bronchodilator, and a separate analysis using FEV_1 _as a percent of predicted, both pre- and post-bronchodilator. We calculated the deaths per 1,000 person years of follow-up for our key covariates. Cox proportional hazard regression models were developed using the SUDAAN procedure SURVIVAL to account for differential follow up in cohort participants. Time of follow up was used as the underlying time metric. Censoring occurred at the date of death certificate or date the participant was last known to be alive. Plots of the log-log survival curves for each covariate were produced to evaluate the proportional hazards assumptions. Age, sex, race, smoking status, education level, body mass index and randomization cohort were included in the adjusted models.

## Results

There were a total of 5,887 participants in the Lung Health Study, of whom 721 died by the end of the follow-up period of up to 15 years. The major causes of death at follow-up were lung cancer and cardiovascular disease with comparatively fewer deaths due to non-malignant respiratory disease. At baseline, the mean age of the cohort was 48.5 years and the mean FEV_1 _was 74.7%. Of these, we had complete data on 5,307 who participated in the examination at year 1 (there were 13 deaths before the year 1 visit). Among the 5,307 on whom we had complete data at year 1, we had 65,472 person years of follow-up, with a median and maximum follow-up time of 12.8 and 14.0 years, and 628 deaths (Table 1). Among the 5,320 on whom we had complete data at year 5 (there were 149 deaths prior to the year 5 visit), we had 45,808 person years of follow-up, with a median and maximum follow-up time of 8.8 and 10.0 years, and 500 deaths (Table 2).

**Table 1:** Characteristics of Analyzed Population at Year 1 (n = 5,307) with 628 deaths at up to 15 years of follow-up

	N	%	Person-Years of Follow-up	Deaths per 1,000 Person Years
Sex				
Female	1993	37.6	24,764	8.3
Male	3314	62.4	40,708	10.4
Age Group, Years				
35-39	522	9.8	6,610	3.8
40-49	2099	39.6	26,350	5.5
50-59	2494	47.0	30,267	13.7
60+	192	3.6	2,245	18.7
Body Mass Index, kg/m^2^				
< 25	2528	47.6	31,168	9.5
25- 30	2082	39.2	25,791	9.1
> = 30	697	13.1	8,513	11.5
Education, Years				
< 12	632	11.9	7,720	13.0
12	1592	30.0	19,629	10.0
> 12	3083	58.1	38,123	8.7
Race				
White	5108	96.3	63,117	9.2
Black	199	3.7	2,355	20.8
Smoking Status				
Former	1439	27.1	17,966	7.0
Intermittent	628	11.8	7,752	9.5
Current	3240	61.1	39,754	10.8
Randomization Group				
Intervention, Ipratroprium	1793	33.8	22,141	8.9
Intervention, Placebo	1785	33.6	22,108	9.0
Control	1729	32.6	21,223	11.0
Stage (pre-bronchodilator)*				
Stage 3 or 4	67	1.3	799	15.0
Stage 2	3432	64.7	42,208	10.7
Stage 1	1354	25.5	16,852	7.3
Restricted	69	1.3	840	9.5
Normal	385	7.3	4,773	6.7
Stage (post-bronchodilator)*				
Stage 3 or 4	19	0.4	224	13.4
Stage 2	2540	47.9	31,126	12.1
Stage 1	1565	29.5	19,386	7.8
Restricted	125	2.4	1,487	12.1
Normal	1058	19.9	13,249	5.8

*Modified chronic obstructive pulmonary disease stage, as defined in methods

**Table 2:** Characteristics of Analyzed Population at Year 5 (n = 5,320) with 500 deaths at up to 10 years of follow-up

	N	%	Person-Years of Follow-up	Deaths per 1,000 Person Years
Sex				
Female	1,992	37.4	17,221	9.4
Male	3,328	62.6	28,587	11.8
Age Group, years				
35-39	25	0.5	214	9.3
40-49	1,708	32.1	14,995	4.7
50-59	2,436	45.8	20,954	11.4
60+	1,151	21.6	9,644	19.7
Body Mass Index, kg/m2				
< 25	2,536	47.7	21,849	10.7
25- 30	2,098	39.4	18,075	10.6
> = 30	686	12.9	5,884	12.7
Education (years)				
< 12	639	12.0	5,496	14.0
12	1,595	30.0	13,735	11.5
> 12	3,086	58.0	26,577	10.0
Race				
White	5,136	96.5	44,226	10.6
Non-White	184	3.5	1,582	19.6
Smoking Status				
Former	1,386	26.1	12,063	7.5
Intermittent	627	11.8	5,403	10.2
Current	3,307	62.2	28,342	12.5
Randomization Group				
Intervention, Ipratroprium	1,791	33.7	15,470	9.6
Intervention, Placebo	1,770	33.3	15,271	10.5
Control	1,759	33.1	15,067	12.7
Stage (pre-bronchodilator)*				
Stage 3 or 4	341	6.4	2,831	21.5
Stage 2	3,587	67.4	30,875	11.3
Stage 1	995	18.7	8,674	7.3
Restricted	92	1.7	768	15.6
Normal	305	5.7	2,659	5.6
Stage (post-bronchodilator)*				
Stage 3 or 4	183	3.4	1,475	29.8
Stage 2	3,048	57.3	26,237	12.0
Stage 1	1,242	23.3	10,775	7.2
Restricted	126	2.4	1,059	13.2
Normal	721	13.6	6,262	7.8

*Modified chronic obstructive pulmonary disease stage, as defined in methods

Table 1 provides additional detail on the covariates of the cohort at year 1, including the total follow-up time and the mortality rate per 1,000 person-years of follow-up. As would be expected, age was the strongest predictor of mortality. Similar data for the Year 5 cohort is displayed in Table 2.

Changes in the COPD classification stages between pre- and post-bronchodilator lung function measurements for the year 1 and the year cohort is shown in Table 3. At year 1, 3,804 of 5,307 (71.7%) remained in the same category for both pre- and post-bronchodilator FEV_1_and at year 5, 4,079 of 5,320 (76.7%) remained in the same category for both pre- and post-bronchodilator FEV_1_. The mean FEV_1_, as a percentage of predicted, increased from 74.1% (Standard deviation [SD] 10.3%) to78.1% (SD 10.0%) at year 1 and from 70.3% (SD 12.5%) to 74.3% (SD 12.1%) at year 5.

**Table 3:** Comparison of pre- and post-bronchodilator classifications from Year 1 and Year 5 visits

Year 1	Post-Bronchodilator					
Pre-Bronchodilator	Stage 3 or 4*	Stage 2	Stage 1	Restricted	Normal	Total
Stage 3 or 4*	17	50	0	0	0	67
Stage 2	1	2442	578	76	335	3432
Stage 1	0	28	959	0	367	1354
Restricted	1	10	0	44	14	69
Normal	0	10	28	5	342	385
Total	19	2540	1565	125	1058	5307
Year 5						
		Post-Bronchodilator				
Pre-Bronchodilator	Stage 3 or 4*	Stage 2	Stage 1	Restricted	Normal	Total
Stage 3 or 4*	172	168	0	1	0	341
Stage 2	10	2823	482	67	205	3587
Stage 1	0	27	748	0	220	995
Restricted	1	18	1	56	16	92
Normal	0	12	11	2	280	305
Total	183	3048	1242	126	721	5320

The rows represent the pre-bronchodilator values and the columns represent the post-bronchodilator values (i.e. at year one 67/5307 were stage 3 or 4 pre- and 19/5307 were stage 3 or 4 post-bronchodilator). Year 1 Post-Bronchodilator

*Modified chronic obstructive pulmonary disease stage, as defined in methods

The Cox proportional hazards models for the year 1 cohort are shown in Table 4. Age, sex, education level, race, and smoking status were significant predictors of mortality, but in these models COPD classification stage reached statistical significance in only stage 2 of the post-bronchodilator model. The area under the curve, from the PROC logistic model, was 69.2% for the pre- and 69.6% for the post- bronchodilator model. In parallel models that used pre- and post-bronchodilator FEV_1_, as a percentage of predicted, a higher FEV_1 _% predicted lower mortality in both the pre- (HR 0.87, 95% CI 0.81, 0.94 per 10% increase) and post-bronchodilator (HR 0.84, 95% CI 0.77, 0.90) models. The area under the curve for the respective models was 69.2% and 69.4%.

**Table 4:** Results from Cox Proportional hazards survival models on year 1 sample- Mortality follow-up of up to 15 years

	Hazards Ratio	95% Confidence Interval
Sex		
Male	1.34	(1.12, 1.59)
Female	1.00	
Age	1.09	(1.07, 1.10)
Body Mass Index	1.00	(0.98, 1.03)
Education		
< 12 Years	1.29	(1.03, 1.61)
12 Years	1.08	(0.90, 1.29)
> 12 Years	1.00	
Race		
White	1.00	
Black	2.13	(1.57, 2.88)
Smoking Status		
Former Smoker	0.64	(0.52, 0.79)
Intermittent Smoker	0.89	(0.69, 1.15)
Current Smoker	1.00	
Randomization Group		
Intervention, Ipratroprium	0.92	(0.76, 1.12)
Intervention, Placebo	0.89	(0.74, 1.09)
Control	1.00	
Stage (Pre-bronchodilator)*		
Stage 3 or 4	1.51	(0.75, 3.03)
Stage 2	1.36	(0.95, 1.95)
Stage 1	0.97	(0.65, 1.44)
Restricted	1.12	(0.51, 2.48)
Normal	1.00	

Stage (Post-bronchodilator)*		
Stage 3 or 4	1.45	(0.41, 5.15)
Stage 2	1.54	(1.20, 1.98)
Stage 1	1.06	(0.80, 1.40)
Restricted	1.63	(0.96, 2.75)
Normal	1.00	

Results for post-bronchodilator lung function measurement is displayed without showing results for the other covariates.

*Modified chronic obstructive pulmonary disease stage, as defined in methods

Similar models for the year 5 follow-up data are shown in Table 5. The main difference seen between the year 1 and year 5 models is that the latter now show an increased risk of Stage 3 or 4 COPD on mortality in both the pre- (HR 2.68, 95% CI 1.51, 4.75) and post-bronchodilator (HR 2.46, 95% CI 1.63, 3.73) models. The area under the curve was 69.0% for the pre- and 69.5% for the post-bronchodilator models. Similar models using FEV_1 _showed that a higher FEV_1 _% predicted lower mortality in both the pre-bronchodilator (HR 0.84, 95% CI 0.75, 0.87 per 10% increase) and post-bronchodilator (HR 0.78, 95% CI 0.73, 0.90) models. The area under the curve for the respective models was 69.4% and 69.8%.

**Table 5:** Results from Year 5 sample, mortality follow-up of up to 10 years

	Hazards Ratio	95% Confidence Interval
Sex		
Male	1.36	(1.12, 1.64)
Female	1.00	
Age	1.09	(1.07, 1.10)
Body Mass Index	1.01	(0.98, 1.03)
Education		
< 12 Years	1.24	(0.96, 1.59)
12 Years	1.10	(0.90, 1.34)
> 12 Years	1.00	
Race		
White	1.00	
Black	1.77	(1.22, 2.56)
Smoking Status		
Former Smoker	0.63	(0.50, 0.80)
Intermittent Smoker	0.85	(0.64, 1.13)
Current Smoker	1.00	
Randomization Group		
Intervention, Ipratroprium	0.86	(0.69, 1.07)
Intervention, Placebo	0.91	(0.74, 1.13)
Control	1.00	
Stage (Pre-bronchodilator)*		
Stage 3 or 4	2.68	(1.51, 4.75)
Stage 2	1.60	(0.94, 2.69)
Stage 1	1.12	(0.63, 1.98)
Restricted	2.25	(1.04, 4.86)
Normal	1.00	

Stage (Post-bronchodilator)*		
Stage 3 or 4	2.46	(1.63, 3.73)
Stage 2	1.11	(0.82, 1.51)
Stage 1	0.74	(0.52, 1.06)
Restricted	1.36	(0.74, 2.47)
Normal	1.00	

*Modified chronic obstructive pulmonary disease stage, as defined in methods

## Discussion

This analysis examined data from the Lung Health Study to determine whether post-bronchodilator lung function predicts mortality. Overall, we found that the pre- and post-bronchodilator measures of lung function, whether used categorically (as stages of COPD) or continuously (as FEV_1 _% predicted) predicted mortality similarly. This finding suggests that post-bronchodilator lung function data may not be needed for studies that look at long term outcomes in COPD.

Most guidelines defining COPD say that spirometry should be performed after the administration of an adequate dose of an inhaled bronchodilator in order to minimize variability [4,21]. These same guidelines, however, state that "neither bronchodilator nor oral glucocorticosteroid reversibility testing predicts disease progression, whether judged by decline in FEV_1_, deterioration of health status, or frequency of exacerbations in patients with a clinical diagnosis of COPD and abnormal spirometry. Small changes in FEV_1 _(e.g., < 400 ml) after administration of a bronchodilator do not reliably predict the patient's response to treatment" [4]. Others have suggested that one cannot use prebronchodilator lung function to define COPD, the reason being that airflow limitation can be variable and that this component can be easily reverse with a bronchodilator [22]. Other research, though, has suggested that bronchodilator responsiveness is highly variable and that "over half the patients initially classified as reversible by the ATS/GOLD definition would be reclassified had they attended on another occasion" [23].

In population-based studies, one would expect that post-bronchodilator lung function measurement would reduce the prevalence of COPD. For example, in the PLATINO study, bronchodilator testing reduced the overall prevalence of FEV_1_/FVC% < 0.70 from 21.7% to 14% [24]. In our analysis the prevalence of severe COPD was lower in the post- compared to the pre-bronchodilator lung function in both the year 1 (0.4% vs. 1.3%) and the year 5 (3.4% vs. 6.4%) cohorts. The finding of a lower prevalence, however, does not necessarily mean that this is the correct prevalence.

Others have looked at this problem in different ways. For example, Hansen et al studied 985 patients with COPD and found that the response to a bronchodilator was a positive prognostic factor along with FEV_1 _at baseline. However, if baseline FEV_1 _was substituted with post-bronchodilator FEV_1_, the bronchodilator reversibility became nonsignificant [25]. Compared to our population, that population had much lower lung function (mean FEV_1 _38.5% of predicted compared to our mean FEV_1 _of 74.7%) and was much older (mean age 61.8 years at baseline compared to our mean age of 48.5 years). Still, the predictive value for FEV_1 _in their study was similar in the pre- (relative risk [RR] 0.60, 95% CI 0.54, 0.81) and post- bronchodilator (RR 0.62, 95% CI 0.56, 0.69) models.

Burrows acknowledged the complexity of the relation between bronchodilator responsiveness and outcomes in obstructive lung disease [26]. He noted that different studies had varying results [27,28] and suggested that several factors, such as how baseline lung function is determined, how responsiveness is measured, and the prevalence of "asthma" in the studied population, may be important determinants of outcomes. His conclusion that mortality is "related to age and to a low initial post-bronchodilator FEV_1_" provides, in part, the historic rationale for using post-bronchodilator lung function to define COPD.

This study has limitations that are important to its interpretation. The most important was that it was not a true "population-based" study but was a clinical intervention trial that targeted early COPD. Study participants had to be current smokers at entry with lung function that was mildly abnormal, and subjects who regularly used bronchodilators were excluded. Although asthma history was not a specific exclusion criterion, excluding people with regular bronchodilator use had the net effect of eliminating subjects with clinically significant asthma from the population. Thus, these findings may not necessarily apply to a population that includes never smokers or where a large proportion of the population has asthma that is symptomatic. Also, a more inclusive population of smokers where reversibility is more common may have yielded different results. This limitation is decreased by our study design that looked at data from the year 1 and year 5 follow-up, at which point some subjects had stopped smoking and many developed symptoms consistent with asthma or COPD. In addition, one would not expect the post-bronchodilator FEV_1 _of never smokers (in the absence of asthma) to differ significantly from the pre-bronchodilator value. Finally, the dose of bronchodilator used in this study (two inhalations, 200-μg total dose, of isoproterenol) is less than what has been used in other clinical trials, some of which have used 400 μg of isoproterenol and 400 μg of albuterol [29]. Thus, it is unknown whether the findings would be similar if a "maximal bronchodilitation" protocol was used.

Another limitation was the absence of other important measures of COPD, such as an impaired exercise testing, impaired diffusion capacity or abnormal imaging. Recent work [30-32] in COPD has highlighted that measures other than lung function are important predictors of impaired function and poor outcomes. Lung function remains, however, the primary means of diagnosing and classifying COPD at the present time and this is unlikely to change in the foreseeable future.

## Conclusion

We found that is this cohort both pre- and post-bronchodilator lung function predicted mortality with similar accuracy. This validates the approach taken in a number of long-term studies where only prebronchodilator lung function is available, although studies that include similar data in never smokers and subjects with asthma are needed.

## Competing interests

DMM has served on advisory boards for Boehringer Ingelheim, Pfizer, GlaxoSmithKline, Sepracor, Astra-Zeneca, Novartis and Ortho Biotech and has received research grants from Astra-Zeneca, GlaxoSmithKline, Novartis and Pfizer. This work was sponsored by a research grant from GlaxoSmithKline. EDG and ASB declare no conflicts of interest.

## Authors' contributions

DMM and EDG participated in the design of the study, drafting the manuscript, and interpreting the results. ASB assisted in acquiring the data, drafting the manuscript, and interpreting the results. All authors have read and approved the final manuscript.
